# Matrix Metalloproteinase-2 (MMP-2) Gene Deletion Enhances MMP-9 Activity, Impairs PARP-1 Degradation, and Exacerbates Hepatic Ischemia and Reperfusion Injury in Mice

**DOI:** 10.1371/journal.pone.0137642

**Published:** 2015-09-10

**Authors:** Hiroyuki Kato, Sergio Duarte, Daniel Liu, Ronald W. Busuttil, Ana J. Coito

**Affiliations:** The Dumont-UCLA Transplant Center, Division of Liver and Pancreas Transplantation, Department of Surgery, David Geffen School of Medicine at UCLA, Los Angeles, CA, United States of America; IDIBAPS—Hospital Clinic de Barcelona, SPAIN

## Abstract

Hepatic ischemia and reperfusion injury (IRI) is an inflammatory condition and a significant cause of morbidity and mortality after surgery. Matrix metalloproteinases (MMPs) have been widely implicated in the pathogenesis of inflammatory diseases. Among the different MMPs, gelatinases (MMP-2 and MMP-9) are within the most prominent MMPs detected during liver IRI. While the role of MMP-9 in liver damage has been fairly documented, direct evidence of the role for MMP-2 activity in hepatic IRI remains to be established. Due to the lack of suitable inhibitors to target individual MMPs *in vivo*, gene manipulation is as an essential tool to assess MMP direct contribution to liver injury. Hence, we used MMP-2^-/-^ deficient mice and MMP-2^+/+^ wild-type littermates to examine the function of MMP-2 activity in hepatic IRI. MMP-2 expression was detected along the sinusoids of wild-type livers before and after surgery and in a small population of leukocytes post-IRI. Compared to MMP-2^+/+^ mice, MMP-2 null (MMP-2^-/-^) mice showed exacerbated liver damage at 6, 24, and 48 hours post-reperfusion, which was fatal in some cases. MMP-2 deficiency resulted in upregulation of MMP-9 activity, spontaneous leukocyte infiltration in naïve livers, and amplified MMP-9-dependent transmigration of leukocytes *in vitro* and after hepatic IRI. Moreover, complete loss of MMP-2 activity impaired the degradation of poly (ADP-ribose) polymerase (PARP-1) in extensively damaged livers post-reperfusion. However, the administration of a PARP-1 inhibitor to MMP-2 null mice restored liver preservation to almost comparable levels of MMP-2^+/+^ mice post-IRI. Deficient PARP-1 degradation in MMP-2-null sinusoidal endothelial cells correlated with their increased cytotoxicity, evaluated by the measurement of LDH efflux in the medium. In conclusion, our results show for the first time that MMP-2 gene deletion exacerbates liver IRI. Moreover, they offer new insights into the MMP-2 modulation of inflammatory responses, which could be relevant for the design of new pharmacological MMP-targeted agents to treat hepatic IRI.

## Introduction

Hepatic ischemia and reperfusion injury (IRI) is a pathological condition characterized by an initial hypoxic insult, which is further accentuated by the restoration of blood flow to the compromised organ [1]. Hepatic IRI remains a significant challenge in surgical procedures where the blood supply to liver is temporarily interrupted, including in clinical orthotopic liver transplantation (OLT) [2]. IR-induced damage is the result of complex interactions between circulating leukocytes, vascular endothelium, extracellular matrix (ECM), and a wide range of other inflammatory mediators [3,4].

Matrix metalloproteinase (MMP) are a family of specialized zinc-dependent proteases that have essential roles in defining how cells interact with their surrounding microenvironment [5]. In addition to extracellular matrix (ECM) turnover, MMPs proteolytically activate or degrade a variety of non-matrix subtracts, including cytokines and chemokines, and have regulatory functions in inflammation and immunity [6]. Among the different MMPs, gelatinases (gelatinase A, MMP-2 and gelatinase B, MMP-9) are notably detected in damaged livers post-surgery, including after human liver transplantation [7,8]. MMP-2 is constitutively expressed in naive livers [9,10], whereas MMP-9 is an inducible enzyme chiefly produced by infiltrating leukocytes after hepatic IRI [9,11].

MMP-2 and MMP-9 have similar proteolytic substrate specificities, but not identical, and there is a growing body of evidence suggesting that these gelatinases can have distinct biological roles [12,13,14,15,16]. Additionally, the same MMP depending on the cell or tissue type in which is expressed, or on the nature of the pathological process, can have opposing functions [17]. In this context, it has been demonstrated that MMP-2 gene deletion reduces the atherosclerotic plaque lesion formation in apoE^−/−^ mice [18], and is beneficial in acute myocardial infarction [19], while it exacerbates myocardial inflammation in viral-induced myocarditis [20]. These apparently paradoxical effects can perhaps be explained by observations that MMPs can act on various substrates in a particular tissue [6]. Despite the considerable progress that has been made in understanding the complex functions of MMPs, the choice of which MMPs to target for therapeutic purposes is still uncertain in various pathological conditions [21]. We have demonstrated that MMP-9 facilitates the migration of leukocytes into inflamed livers [11]; nevertheless, the role of MMP-2 in liver IRI remains less well characterized. The current MMP inhibitors suitable for *in vivo* use differ in their inhibitory potencies towards MMPs, but none of these drugs is selective for a given MMP [22]. Therefore, we used MMP-2 null mice and respective wild-type littermates to evaluate the direct contribution of MMP-2 activity to the development of hepatic IRI.

[12,13,14,15,16] Additionally, the same MMP depending on the cell or tissue type in which is expressed, or on the nature of the pathological process, can have opposing functions.[17] In this context, it has been demonstrated that MMP-2 gene deletion reduces the atherosclerotic plaque lesion formation in apoE^−/−^ mice,[18] and is beneficial in acute myocardial infarction,[19] while it exacerbates myocardial inflammation in viral-induced myocarditis.[20] These apparently paradoxical effects can perhaps be explained by observations that MMPs can act on various substrates in a particular tissue.[6] Despite the considerable progress that has been made in understanding the complex functions of MMPs, the choice of which MMPs to target for therapeutic purposes is still uncertain in various pathological conditions.[21] We have demonstrated that MMP-9 facilitates the migration of leukocytes into inflamed livers;[11] nevertheless, the role of MMP-2 in liver IRI remains less well characterized. The current MMP inhibitors suitable for *in vivo* use differ in their inhibitory potencies towards MMPs, but none of these drugs is selective for a given MMP.[22] Therefore, we used MMP-2 null mice and respective wild-type littermates to evaluate the direct contribution of MMP-2 activity to the development of hepatic IRI.

## Materials and Methods

### Mice and Model of Hepatic IRI

C57BL/6 mice carrying the MMP-2-null allele were obtained from Riken, Japan [23], rederived by sterile embryo transfer to surrogate mothers and housed in the UCLA animal facility under specific pathogen-free conditions. A warm hepatic IRI model was performed in 10-week-old male MMP-2^−/−^ mice and matched MMP-2^+/+^ WT littermates, as previously described [11]. Briefly, the arterial and portal venous blood supplies were interrupted to the cephalad lobes of the liver for 90 minutes using an atraumatic clip. After 90 minutes of ischemia the clip was removed, thus initiating hepatic reperfusion. Additionally, separate groups of C57BL6 mice, MMP-2^-/-^ mice, and MMP-9^-/-^ mice were treated with an anti-MMP-2 neutralizing monoclonal antibody (3 mg/kg, i.v.) or a specific PARP-1 inhibitor (PJ34, 20 mg/Kg i.v) at reperfusion. Mice in control groups were injected with isotype-matched IgG or vehicle. Administration of the anti-MMP-2 neutralizing monoclonal antibody to MMP-2 deficient mice did not have an effect on the serum transaminase levels in these mice. The animal studies were carried out with the approval of the University of California, Los Angeles (UCLA) Animal Research Committee (ARC) and followed the guidelines outlined in the "Guide for the Care and Use of Laboratory Animals" prepared by the National Academy of Sciences and published by the National Institutes of Health.

### Assessment of Liver Damage

Serum alanine transaminase (ALT) and serum aspartate transaminase (AST) levels were measured using a commercially available kit (Teco Diagnostics, Anaheim, CA), following manufacturer's instructions. Liver specimens were fixed with a 10% buffered formalin solution, embedded in paraffin and processed for H&E staining.

### Myeloperoxidase (MPO) Assay

Myeloperoxidase activity was evaluated in frozen tissue homogenized in an iced solution of 0.5% hexadecyltrimethyl-ammonium and 50 mmol/L of potassium phosphate buffer solution with pH adjusted to 5. After centrifugation, the supernatants were mixed in a solution of hydrogen peroxide-sodium acetate and tetramethyl benzidine (Sigma). The quantity of enzyme degrading 1 μmol/L of peroxide/minute at 25°C per gram of tissue was defined as 1U of MPO activity.

### Immunohistochemistry

Immunostaining was performed in cryostat sections, as described [9,11]. Mac-1 (M1/70), Ly-6G (1A8), and PECAM-1 (MEC13.3) from BD Biosciences, MMP-2 (Ab19167; Millipore), and MMP-9 (AF909; R&D Systems) antibodies were used at optimal dilutions. Sections were blindly evaluated by counting ten HPFs/section in triplicates. Dual/triple staining was detected by immunofluorescence with Alexa Fluor 594-red anti-rabbit IgG (H+L) and Alexa Fluor 488-green anti-rat IgG (H+L) (Molecular Probes); Vectashield mounting media with DAPI (Vector Laboratories) was used for nuclear staining. Slides were analyzed using a Leica Confocal Microscope.

### Western blot and Zymography Analysis

Western blots and zymography were performed as described [9,11]. Briefly, proteins (40 μg/sample) in sodium dodecyl sulfate (SDS)-loading buffer were electrophoresed through 10–18% SDS-polyacrylamide gel electrophoresis (PAGE) and transferred to PVDF membranes. Membranes were incubated with specific antibodies against PARP-1 (#9542, and #5625, Cell Signaling Technology). After development, membranes were stripped and reblotted with anti-actin antibody (Santa Cruz Biotechnology).

Gelatinolytic activity was detected in liver extracts (80μg) by 10% SDS-PAGE contained 1mg/ml of gelatin (Invitrogen), under non-reducing conditions. After incubation in renaturation buffer (Bio-Rad) and development buffer (50 mmol/L Tris-HCl, 5 mmol/L CaCl2, and 0.02% NaN3, pH 7.5), gels were stained with Coomassie brilliant blue R-250 (Bio-Rad), and destained with methanol/acetic acid/water (20:10:70). Prestained molecular weight markers (Bio-Rad), MMP-2, and MMP-9 (BIOMOL International) served as standards. Relative quantities of protein were determined using a densitometer (NIH Image J software)

### RNA Extraction and Reverse Transcriptase PCR

RNA was extracted from livers with Trizol (Life Technologies), as described [11]. Reverse transcription was performed using 5 μg of total RNA in a first-strand cDNA synthesis reaction with SuperScript III RNaseH Reverse Transcriptase (Life Technologies), as recommended by the manufacturer. The cDNA product was amplified by PCR using primers specific for each target cDNA.

### Isolation and Culture of Mouse Cells

Isolation of neutrophils and sinusoidal endothelial cells (SEC) from MMP-2^+/+^ and MMP-2^-/-^ mice was performed according to previously published methods [11,24]. Briefly, to isolate neutrophils, femurs and tibias were harvested and stripped of all muscle and sinew, and bone marrow was flushed with 2.5 ml of RPMI-1640 containing 5% fetal calf serum on ice. Cells were pelleted and erythrocytes were removed by hypotonic lysis. The entire bone marrow preparation was resuspended in Hanks’ balanced saline solution and cells were layered on a Percoll (Sigma–Aldrich) gradient (3 ml of 55%, top; 3 ml of 65%, middle; 4 ml of 80% Percoll) and centrifuged at 2000 rpm for 30 minutes at 10°C. Mature neutrophils were recovered at the interface of the 65% and 80% fractions and were >90% pure and >95% viable in the neutrophil-rich fraction as determined by Ly-6G immunostaining/morphology and trypan blue exclusion, respectively. To isolate SECs, anesthetized mice were subject to a midline laparotomy and cannulation of the inferior vena cava (IVC) followed by liver perfusion with an EDTA-chelating perfusion buffer (10mM Hepes, 0.15M NaCl, 0.42g/L KCl, 0.99g/L Glucose, 2.1g/L NaHCO3, 0.19g/L EDTA). After perfusion with collagenase buffer (50 mM Tris–HCl, pH 7.5, 150 mM NaCl, 5 mM CaCl2 and 0.02% Brij-35), livers were minced and cells dispersed in culture medium; nonparenchymal cells were separated from hepatocytes by low-speed centrifugation methods. SECs were then purified using a two-step Percoll gradient (25/50%) and selective adherence. SECs were cultured on 24-well collagen-coated plates (BD BioCoat) in RPMI-1640 medium with 2% fetal bovine serum (FBS). SECs were incubated in the presence or absence of MMP-2 inhibitor-III (100 or 500nM, Calbiochem) and PARP inhibitor Pj34 (50uM). The cell lysates were prepared for mRNA and protein evaluations, and the supernatants were collected for LDH enzyme assays.

### LDH Enzyme Assay

The release of lactate dehydrogenase (LDH) in culture media was assessed with the LDH Cytotoxicity Assay kit (Cayman Chemical), according to the manufacturer’s instructions.

### *In Vitro* Migration Assay

Isolated neutrophils were resuspended in RPMI-1640 without FBS, and transmigration through fibronectin was performed using a commercially available in vitro cell migration assay kit (BD Bioscience), as previously described [11]. Briefly, 6.5-mm-diameter Transwell inserts with 3-μm pores were used; these inserts were either coated with fibronectin or uncoated (control invasion chambers) on 24-well culture trays. Neutrophils were added at 5×10^5^ cells/well to the top chamber with or without MMP-9 inhibitor-I (100nM, Calbiochem); fMLP was added to the lower chamber. Neutrophils were incubated at 37°C and 5% CO2 for 4h, and the cells that had migrated into the lower chamber were collected, stained, and counted.

### Data Analysis

Data in the text and figures are expressed as mean ± standard deviation. Statistical comparisons between groups of normally distributed data were performed with the Student t-test using statistical package SPSS (SPSS Inc., Chicago, IL). Kaplan-Meier analysis was used to determine statistical significance of the differences in mouse survival. P values of less than 0.05 were considered statistically significant.

## Results

### Patterns of MMP-2 Expression in Wild-Type Livers

**Fig 1:**
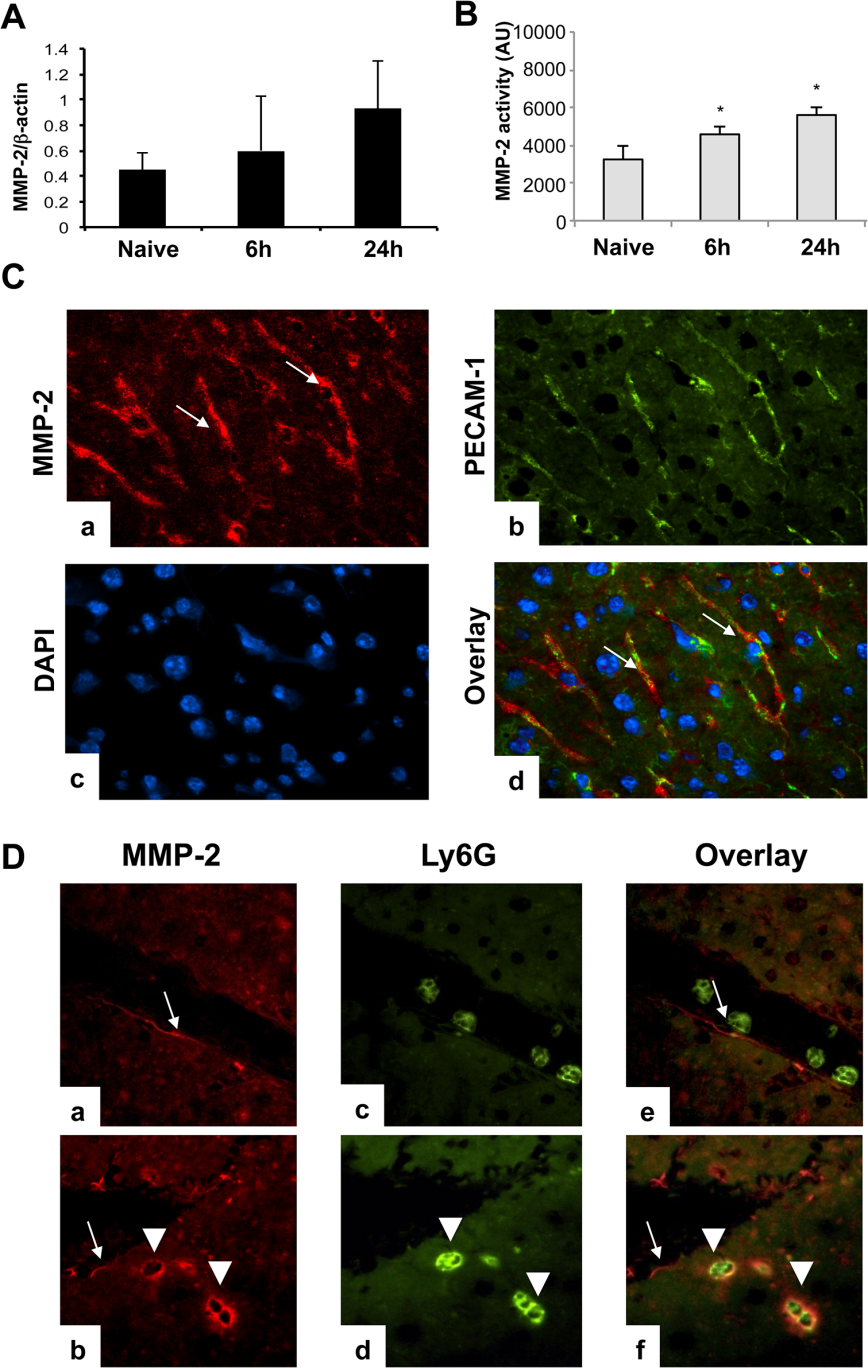
Time course and sources of MMP-2 expression in MMP-2^+/+^ livers. MMP-2 mRNA expression (panel A) and MMP-2 activity (panel B) were readily detected in wild-type livers before and after hepatic IRI. MMP-2 positive staining was detected in the vasculature of naïve MMP-2^+/+^ livers (panel C); MMP-2 in red (a; Alexa Fluor 594), PECAM-1 in green (b; Alexa Fluor 488), nuclear stain in blue (c; Dapi), and staining overlay (d). In addition to the vasculature, MMP-2 positive staining was also detected in Ly6G neutrophils in MMP-2^+/+^ livers at 6h post-IRI (panel D); MMP-2 in red (a and b), Ly6G in green (c and d) and staining overlay (e and f); (arrows and arrow heads denote MMP-2 positive staining in the liver vasculature and in infiltrating leukocytes, respectively; n = 5/group; *p<0.05 relative to naïve livers).

MMP-2 mRNA expression and enzymatic activity were eagerly detected in wild-type livers before (naïve livers) and after IRI. Moreover, MMP-2 activity was modestly upregulated in livers after surgery (Fig 1A and 1B). In naïve wild-type livers, there was significant colocalization of the MMP-2 protein expression and the endothelial cell marker PECAM-1 (Fig 1C). In damaged wild-type livers after IRI, while MMP-2 expression was scarcely noticed in parts of the vascular bed, it was also detected in a small population of infiltrating leukocytes; double staining showed colocalization of MMP-2 and the neutrophil marker Ly6G in the damaged liver tissue (Fig 1D).

### MMP-2 Deficient Mice Had Increased Liver Damage and Reduced Survival after IRI

**Fig 2:**
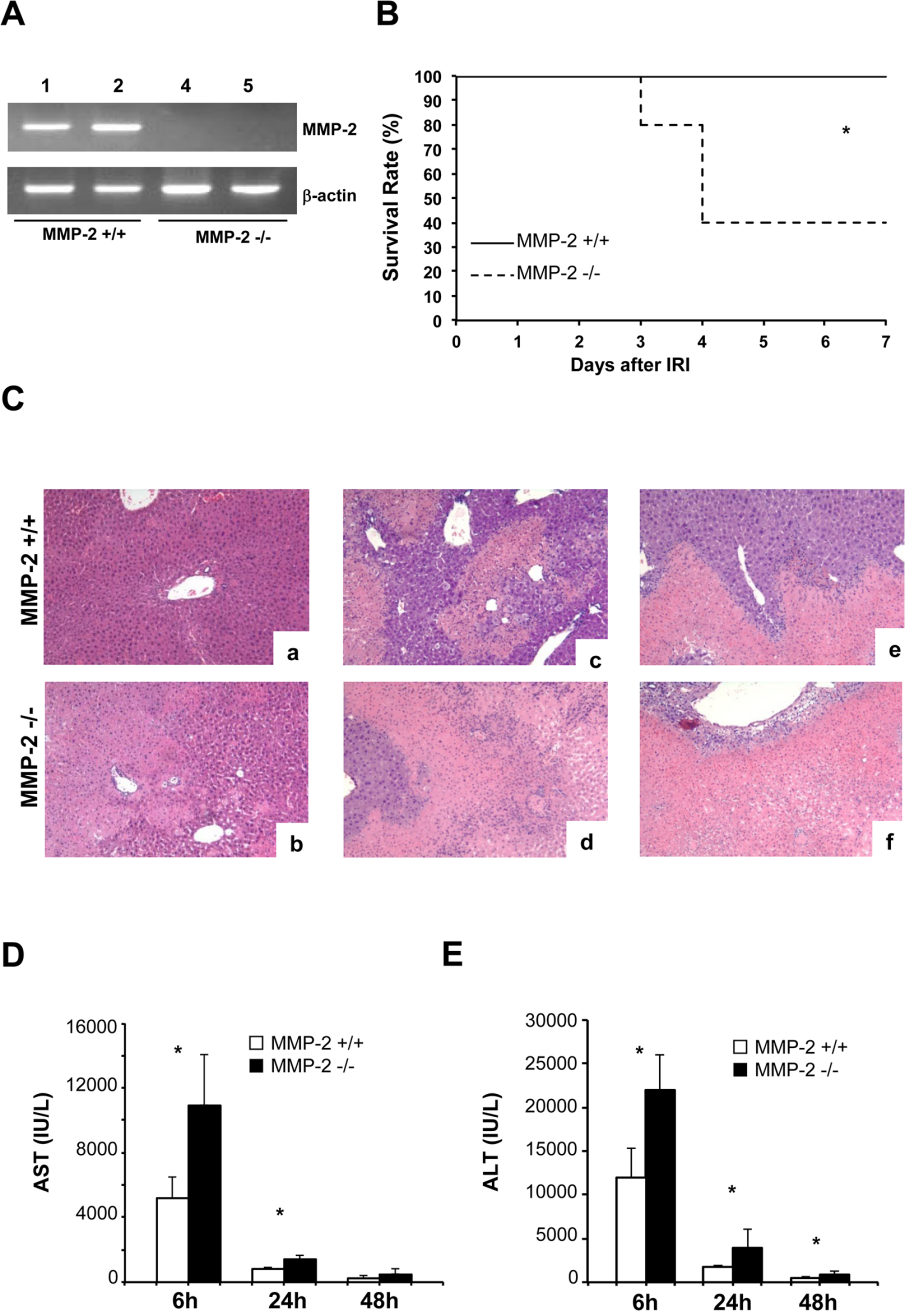
Survival, liver histology and serum transaminases in MMP-2^-/-^ and MMP-2^+/+^ mice after liver IRI. MMP-2 expression (panel A) was detected in MMP-2^+/+^ livers (lanes 1 and 2) and absent in MMP-2^-/-^ livers (lanes 3 and 4) post-IRI. Survival (panel B) of MMP-2^-/-^ deficient mice (dotted line) was significantly reduced at 7 days post-IRI, as compared with MMP-2^+/+^ mice (solid line). Representative H&E staining (panel C) of MMP-2^+/+^ (a, c, and e) and MMP-2^-/-^ (b, d, and f) livers at 6h (a, and b), 24h (c, and d), and 48h (e, and f) post-I/R injury showed that disruption of lobular architecture was significantly exacerbated in MMP-2^-/-^ livers post-reperfusion. The serum transaminase levels, sAST (panel D) and sALT (panel E), were markedly increased in MMP-2^-/-^ mice, particularly at 6h and 24h after IRI; (n = 5–6 mice/group *p<0.05).

To test the significance of MMP-2 expression in liver IRI, our experiments included MMP-2 deficient mice, which showed no detectable MMP-2 activity, and respective wild-type (MMP-2^+/+^) control mice (Fig 2A). MMP-2 deficiency resulted in animal loss; 3 out of 5 MMP-2^-/-^ mice failed to recover from injury and succumbed after 48h post-reperfusion. In contrast, all MMP-2^+/+^ (WT) animals survived 7 days post-IRI, (Fig 2B). Wild-type livers showed significant sinusoidal congestion and necrosis after reperfusion, but MMP-2 deficiency led to considerably increased necrosis and further lobular architecture disruption at 6h, 24h, and 48h post-IRI (Fig 2C). The serum aspartate aminotransferase (AST) and alanine aminotransferase (ALT) (U/L) were also significantly increased in MMP-2^-/-^ mice at 6h, 24h and 48h post-IRI (Fig 2D and 2E). All together, these results show that loss of MMP-2 activity exacerbated hepatic IRI.

### Deficiency in MMP-2 Amplified Leukocyte Accumulation and Activation in Hepatic IRI

**Fig 3:**
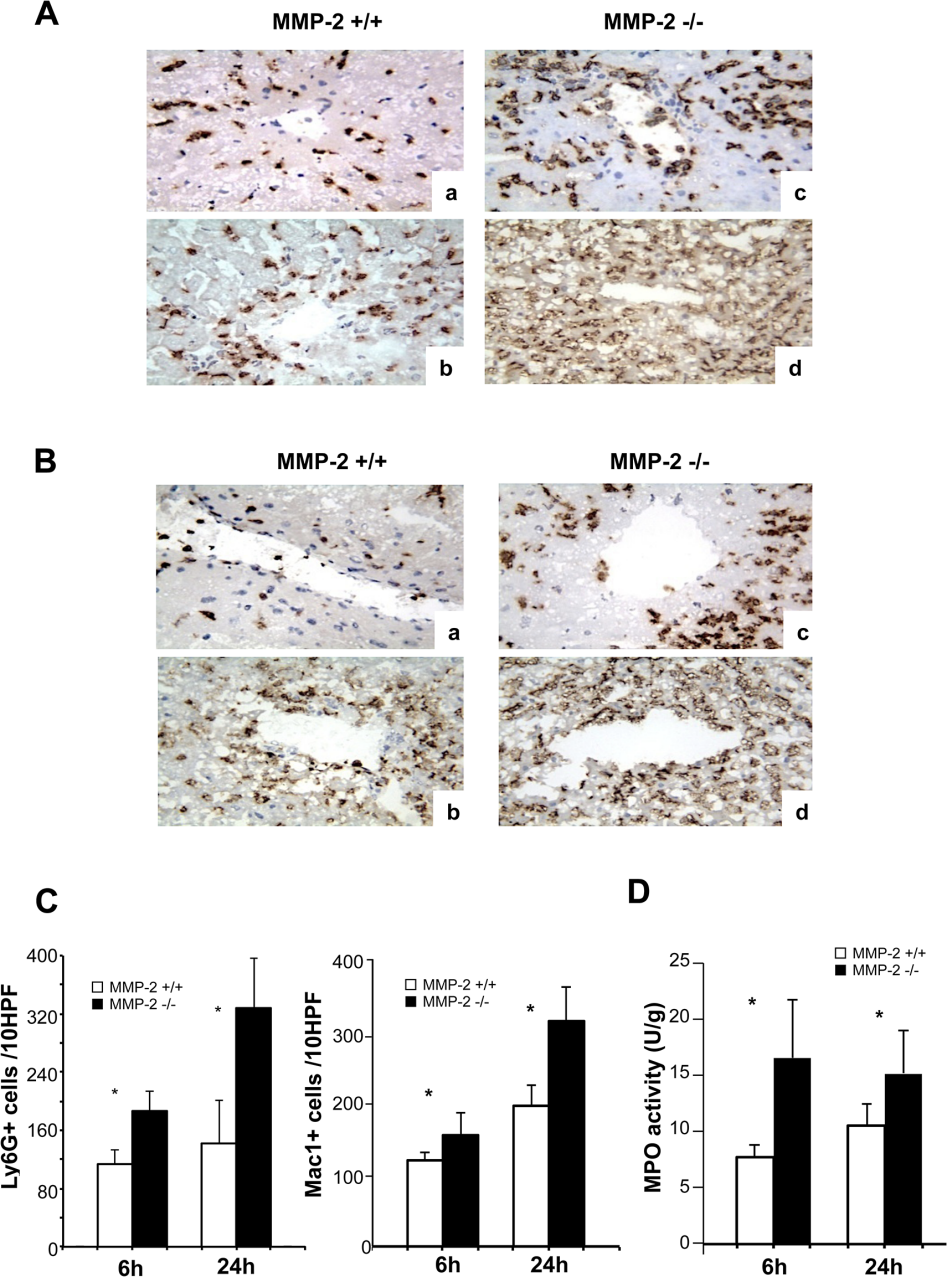
Leukocyte infiltration in MMP-2^-/-^ and MMP-2^+/+^ livers. Representative immunostaining of Ly-6G+ neutrophils (panel A) and Mac-1+ leukocytes (panel B) in MMP-2^+/+^ (a, and b) and MMP-2^-/-^ (c, and d) livers at 6h (a, and c) and 24h (b, and d) post-reperfusion; infiltrating leukocytes were detected in significantly higher numbers in livers of MMP-2^-/-^ mice after IRI (panel C). MPO enzymatic activity (panel D) was also markedly upregulated in MMP-2^-/-^ livers post-IRI (n = 5/group; *p<0.05).

**Fig 4:**
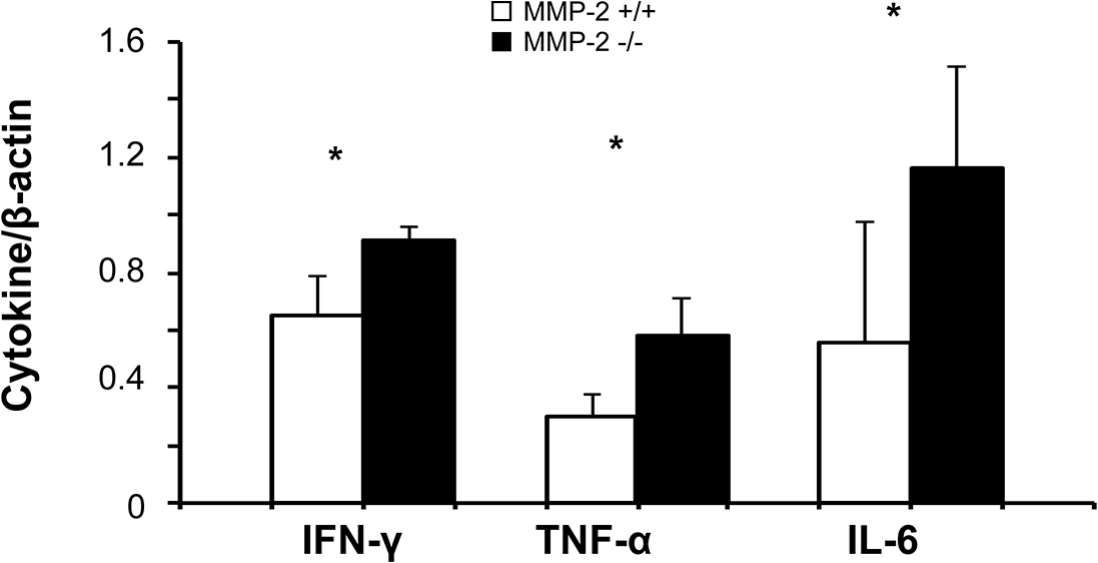
Pro-inflammatory cytokine expression in MMP-2^-/-^ and MMP-2^+/+^ livers. IFN-γ, TNF-α, and IL-6, were upregulated in MMP-2^-/-^ deficient livers post-IRI, compared to respective controls (n = 5/group; *p<0.05).

Leukocytes are critical inflammatory mediators of liver IRI. Ly-6G neutrophils (6h: 187±27 vs. 125±18, 24h: 328±69 vs. 143±60; p<0.05), and Mac-1 leukocytes (6h: 155±31 vs. 123±13, 24h: 315±48 vs. 196±30; p<0.05) and were markedly increased in MMP-2^-/-^ livers post-IRI (Fig 3). Mac-1 is a macrophage differentiation antigen abundantly expressed in stimulated macrophages and present to a lesser degree in granulocytes [25]. Moreover, MPO activity (U/g), which is often used as a neutrophil migration index, was also increased in MMP-2^-/-^ livers at 6h (15.9±5.0 vs. 7.4±1.1; p<0.05) and 24h (14.5±3.7 vs. 10.1±2.0; p<0.05) post-reperfusion, (Fig 3D). The extent of leukocyte infiltration correlated with proinflammatory cytokine expression; IFN-γ (0.91±0.05 vs. 0.65±0.14; p<0.05), TNF-α (0.58±0.13 vs. 0.30±0.08; p<0.05), and IL-6 (1.16±0.35 vs. 0.55±0.42 p<0.05) were all significantly upregulated in MMP-2^-/-^ livers at 6h post-IRI, (Fig 4). Thus, MMP-2 deficiency resulted in massive leukocyte infiltration and activation after liver IRI.

### Anti-MMP-2 Antibody Therapy Worsened Liver Injury

**Fig 5:**
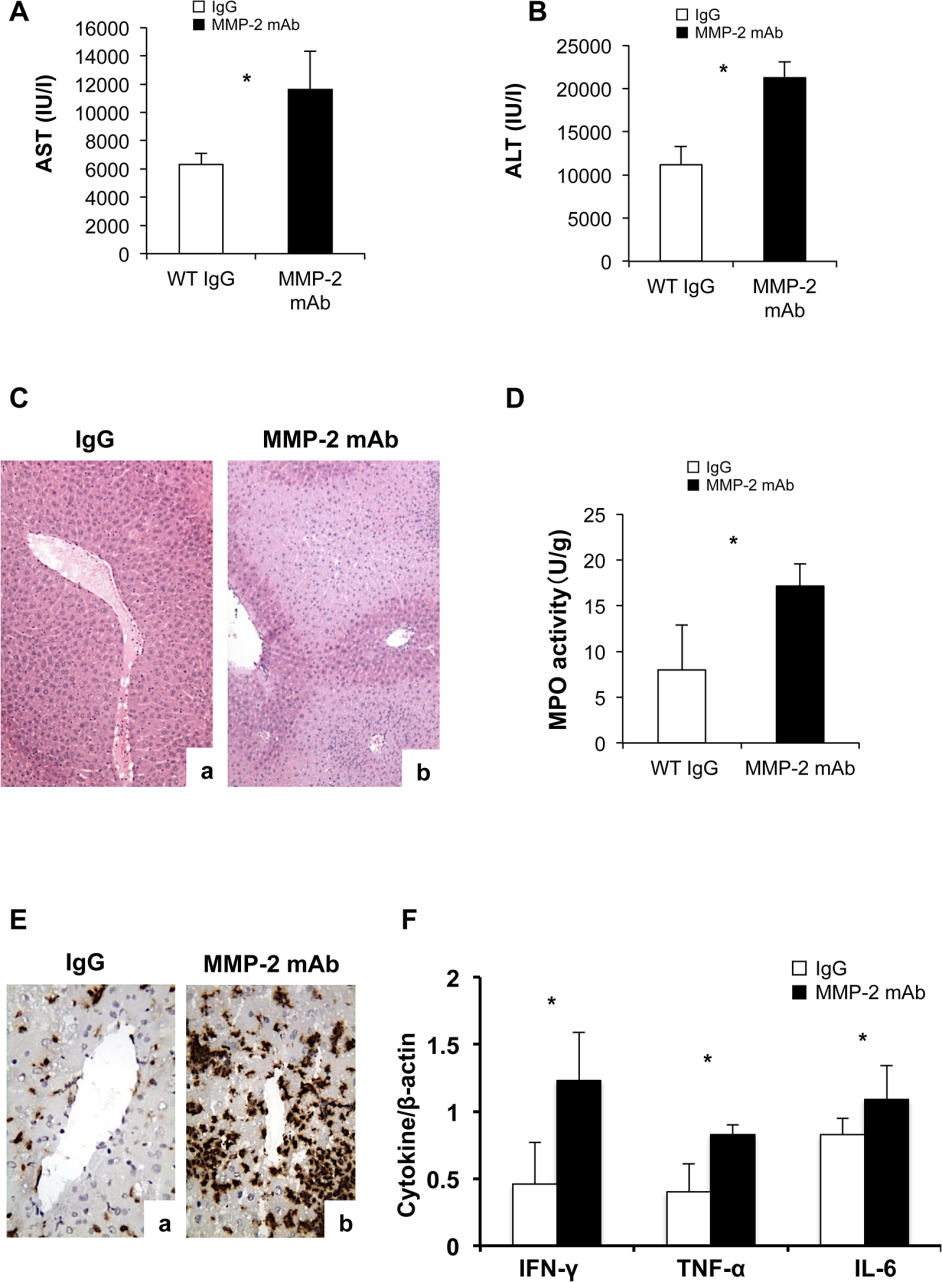
Serum transaminases, liver histology and leukocyte infiltration/ activation in anti-MMP-2 antibody treated mice. AST (panel A) and ALT (panel B) levels were elevated in blood samples of anti-MMP-2 antibody treated mice collected 6h after liver IRI. Liver histological preservation (panel C) was further impaired in anti-MMP-2 antibody treated mice (b), compared to IgG treated controls (a). Moreover, MPO activity (panel D), Mac-1 leukocyte infiltration (panel E) and proinflammatory cytokine expression (panel F) were all increased in anti-MMP-2 antibody treated mice, compared to IgG treated controls (n = 4/group; *p<0.05).

To address potential redundant mechanisms of knockout mice, we carried additional experiments in which wild-type C57BL6 mice were treated with a neutralizing monoclonal antibody against MMP-2 and subjected to hepatic IRI. Compared to IgG-treated controls, mice treated with anti-MMP-2 antibodies were characterized by exacerbated liver damage, increased MPO activity levels, leukocyte infiltration, and enhanced proinflammatory cytokine expression after 6h of reperfusion (Fig 5). These results confirmed a protective role for MMP-2 activity during hepatic IRI.

### MMP-2 Inhibition Upregulated Liver MMP-9 Activity and Increased MMP-9-Dependent Leukocyte Migration

**Fig 6:**
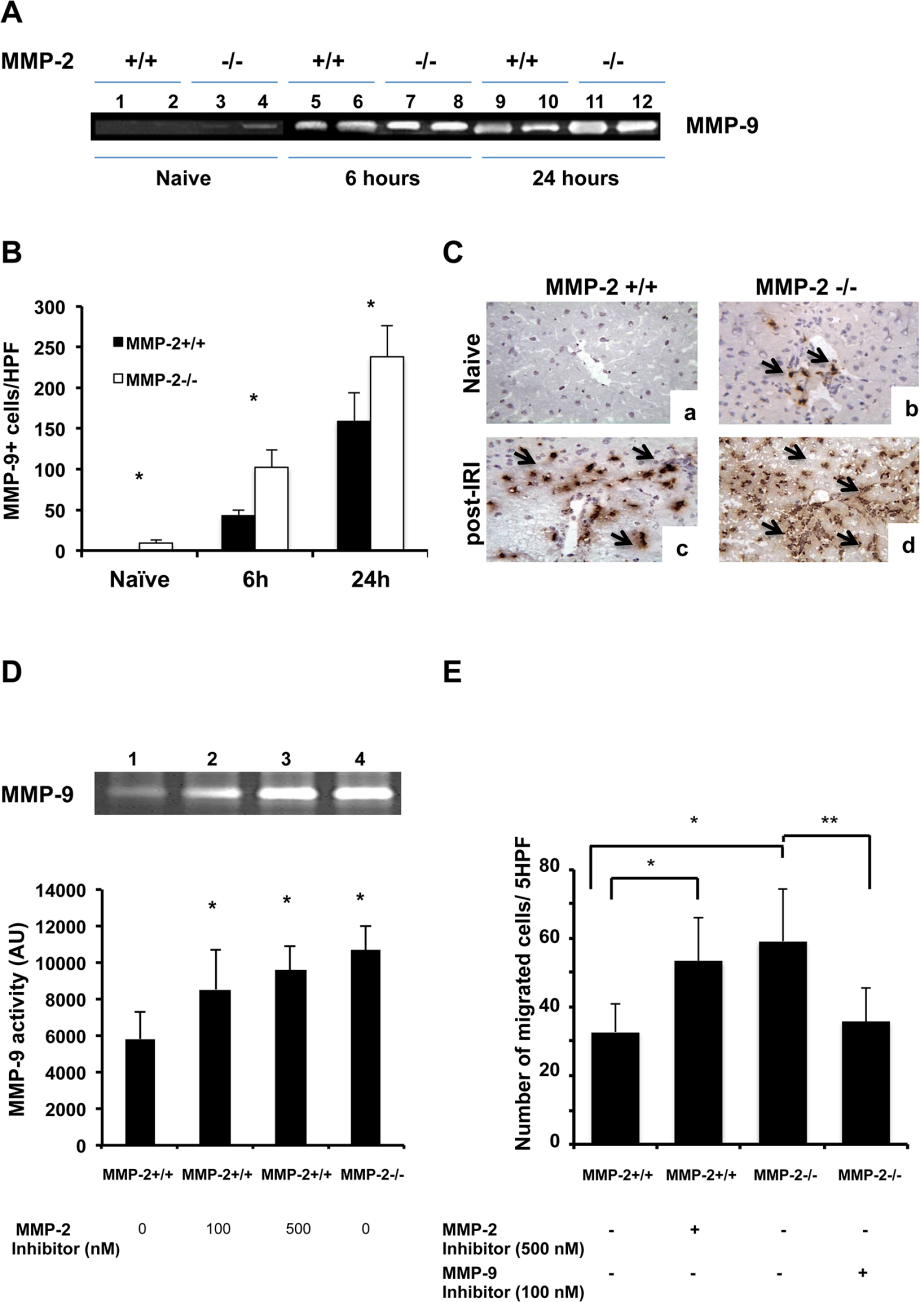
Effect of MMP-2 selective inhibition on MMP-9 activity and MMP-9-dependent leukocyte migration. MMP-9 activity (panel A) was increased in MMP-2^-/-^ livers (lanes 3, 4, 7, 8, 11, and 12), compared to respective MMP-2^+/+^ controls (lanes 1, 2, 5, 6, 9, and 10); naïve livers (lanes 1 to 4), and livers harvested at 6h (lanes 5 to 8) and 24h (lanes 9 to 12) post-IRI. Infiltrating MMP-9+ leukocytes (panel B) were detected in higher numbers in MMP-2^-/-^ livers than in MMP-2^+/+^ livers. Representative immunostaining (panel C) shows that MMP-9+ leukocytes were virtually absent from MMP-2^+/+^ naïve livers (a), but readily detected in MMP-2^-/-^ naïve livers (b); MMP-9+ leukocyte infiltration was also lower in MMP-2^+/+^ livers (c) when compared to MMP-2^-/-^ livers (d) at 6h post-reperfusion. MMP-9 activity (panel D) was significantly increased in isolated MMP-2^+/+^ neutrophils treated with a selective MMP-2 inhibitor in doses of 100nM (lane 2) and 500nM (lane 3) and in MMP-2^-/-^ neutrophils (lane 4), compared to respective MMP-2^+/+^ controls (lane1). Moreover, *in vitro* cell migration (panel E) was significantly enhanced in MMP-2^+/+^ neutrophils treated with a MMP-2 selective inhibitor and in MMP-2^-/-^ neutrophils; on the other hand, the addition of a MMP-9 selective inhibitor to MMP-2^-/-^ neutrophils significantly reduced their migration (n = 5/group; *in vitro* data is expressed as mean ± SD of four independent experiments; *p<0.05 relative to untreated MMP-2^+/+^ samples and **p<0.05 relative to untreated MMP-2^-/-^ samples).

MMP-9 expressed by leukocytes facilitates their migration into injured livers [3]. MMP-2 deletion resulted in a significant spontaneous infiltration of MMP-9+ leukocyte in naïve livers. MMP-2^-/-^ livers were essentially characterized by higher levels of MMP-9 activity (about 2-fold higher) and increased numbers of infiltrating MMP-9+ leukocytes before (9.5±3.7 vs. 0.7±0.4; p<0.05) and after 6h (102.7±21.4 vs. 43.0±7.2; p<0.05) and 24h (238.1±38.0 vs. 158.5±34.7; p<0.05) of IRI (Fig 6A–6C). These observations were further supported *in vitro*; MMP-9 activity was significantly higher in isolated MMP-2-/- neutrophils and in MMP-2^+/+^ neutrophils cultured in the presence of a selective MMP-2 inhibitor, compared to respective MMP-2^+/+^ untreated controls (Fig 6D). *In vitro* migration was also notably enhanced in MMP-2-/- neutrophils and in MMP-2^+/+^ neutrophils treated with the MMP-2 selective inhibitor (Fig 6E). However, treatment with a MMP-9 selective inhibitor of MMP-2^-/-^ neutrophils, which had increased MMP-9 activity (Fig 6D), significantly impaired their ability to migrate (Fig 6E). In brief, MMP-2 inhibition resulted in upregulation of MMP-9 activity and enhanced MMP-9 dependent leukocyte migration.

### Loss of MMP-2 Activity Resulted in Almost Absence of PARP Degradation in Hepatic IRI

**Fig 7:**
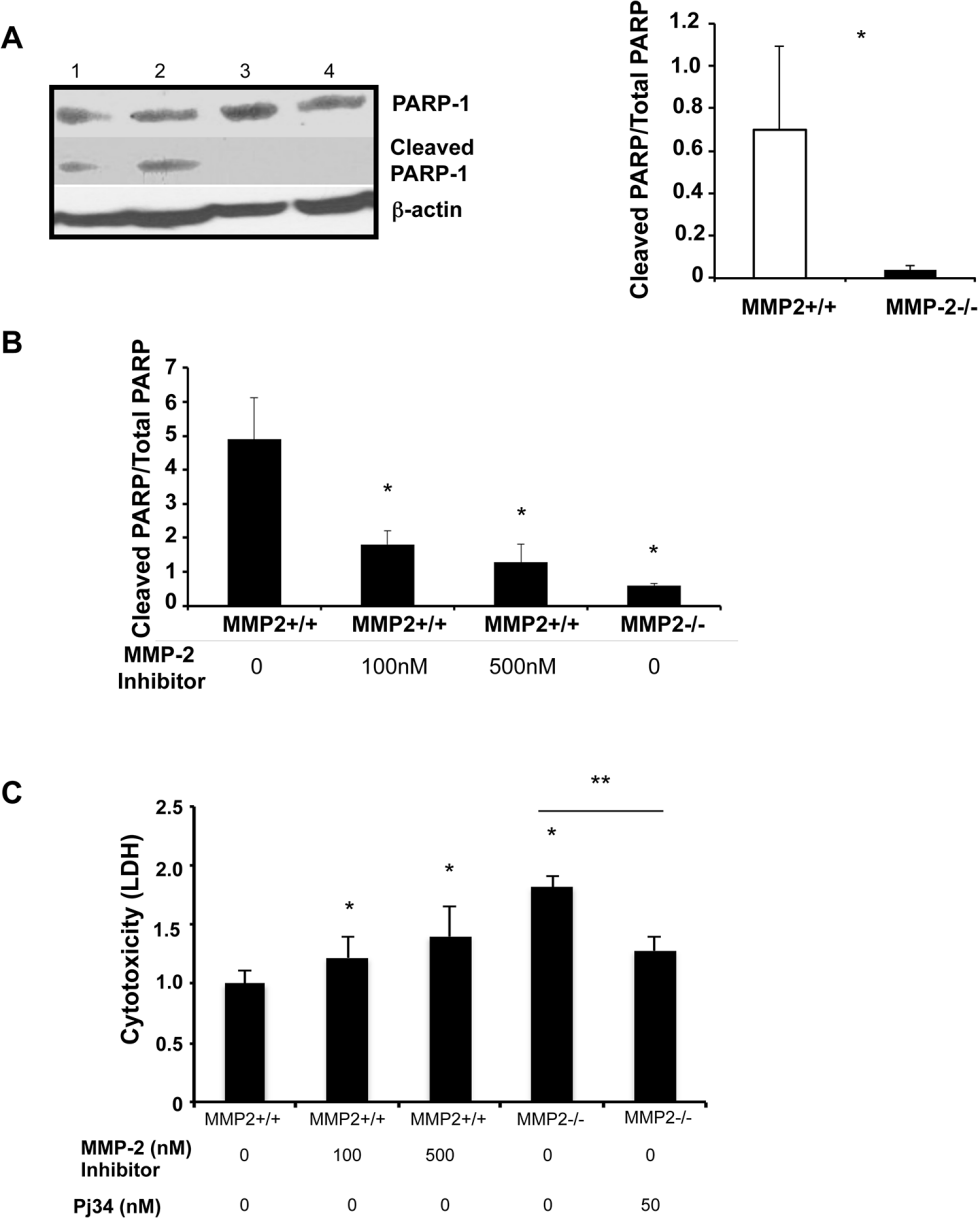
Effect of MMP-2 activity inhibition on PARP-1 degradation. The cleaved form of PARP-1 (panel A) was detected in the MMP-2^+/+^ livers (lanes 1 and 2) and virtually absent in MMP-2^-/-^ livers (lanes 3, and 4) at 6h post-IRI; the cleaved PARP/total PARP ratio was profoundly depressed in the MMP-2^-/-^ livers post-reperfusion. The cleaved PARP/total PARP ratios (panel B) were also markedly decreased in lysates of MMP-2^-/-^ SECs and MMP-2 inhibitor-treated MMP-2^+/+^ SECs. The LDH levels (panel C) were increased in MMP-2^-/-^ SECs and in MMP-2 inhibitor-treated MMP-2^+/+^ SECs, compared with controls (n = 4–5/group; *in vitro* data is expressed as mean ± SD of four independent experiments; *p<0.05 relative to MMP-2^+/+^ samples and **p<0.05 relative to MMP-2^-/-^ samples).

**Fig 8:**
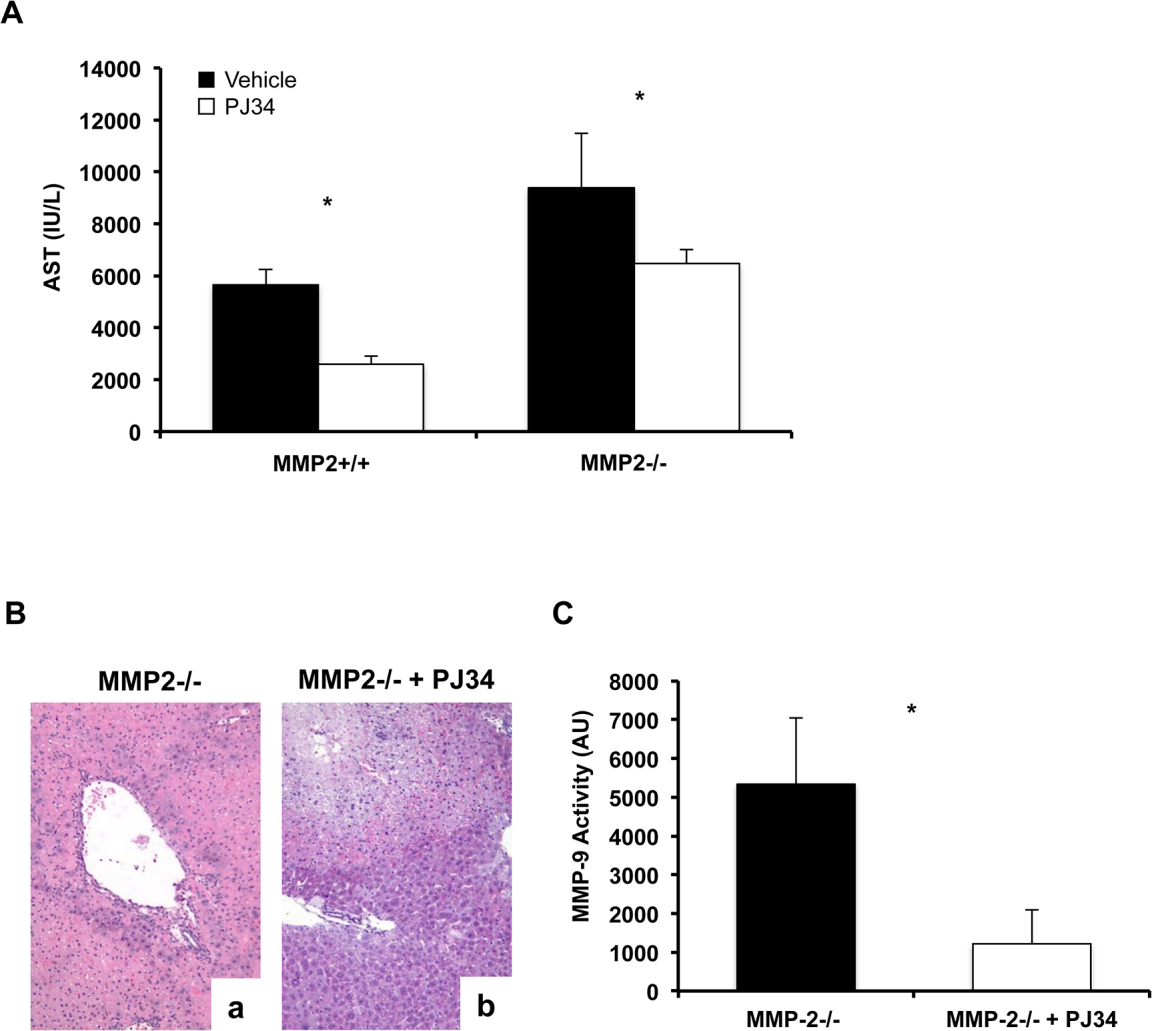
Effect of PJ34 (PARP inhibitor) administration in hepatic IRI. Administration of PJ34 to wild-type mice and to MMP-2^-/-^ mice at reperfusion reduced the serum AST levels (panel A) in these mice at 6h post-IRI. MMP-2^-/-^ mice treated with PJ34 showed improved liver histological preservation (panel B) post-IRI; H&E staining of vehicle treated-MMP-2^-/-^ (a) and PJ34 treated-MMP-2^-/-^ (b) livers post-reperfusion. MMP-9 activity was significantly depressed in MMP-2 null mice treated with PJ34 at 6h after IRI (n = 4–5/group; *p<0.05).

MMP-2 is present in liver nuclear extracts and it cleaves PARP-1 *in vitro* [26]. In our settings, the levels of full-length PARP-1 were relatively higher in MMP-2^-/-^ livers at 6h post-IRI, compared to controls (Fig 7A). Though, cleaved PARP was almost undetectable in MMP-2^-/-^ livers post-reperfusion and the ratio of cleaved PARP/total PARP was profoundly depressed in these livers (~17-fold decrease), providing an indication that MMP-2 also cleaves PARP-1 in liver IRI (Fig 7A). In support, the ratios of cleaved PARP/ total PARP were markedly decreased in lysates of MMP-2^-/-^ sinusoidal endothelial cells (~8-fold decrease) and in lysates of MMP-2^+/+^ sinusoidal endothelial cells treated with a specific MMP-2 inhibitor (dose dependent, ~3–4-fold decrease), (Fig 7B). Impaired PARP-1 cleavage in MMP-2-deficient sinusoidal endothelial cells, and in MMP-2 inhibitor-treated MMP-2^+/+^ sinusoidal endothelial cells, correlated with their increased cytotoxicity, evaluated by the measurement of LDH efflux in the medium, (Fig 7C). Moreover, the addition of a PARP selective inhibitor to cultured MMP-2^-/-^ sinusoidal endothelial cells reduced the LDH levels released by these cells, (Fig 7C). The PARP inhibitor PJ34 has a beneficial effect in several experimental models of ischemia-reperfusion injury [27,28,29], including in our liver IRI model; administration of P34 to wild-type mice at reperfusion lowered the AST levels in these mice post-IRI (Fig 8A). We next tested whether administration of PJ34 ameliorates hepatic IRI in the absence of MMP-2. Indeed, administration of PJ34 to MMP-2^-/-^ deficient mice resulted in a rather modest but still significant protection again hepatic IRI; serum AST levels and histological liver damage were both reduced in PJ34 treated MMP-2-null mice (Fig 8A and 8B). PARP activation can lead to organ dysfunction through multiple pathways. In our settings, the administration of PJ34 to MMP-2 null mice significantly depressed MMP-9 activity post-IRI in these mice (Fig 8C). Thus, our data support a protective PARP-dependent role of MMP-2 activity in hepatic IRI and are in line with the results of a recent study, which showed that PJ34 reduces MMP-9 and protects against traumatic brain injury [30].

### Anti-MMP-2 Antibody-Induced Liver Damage in MMP-9 Null Mice Post-IRI

**Fig 9:**
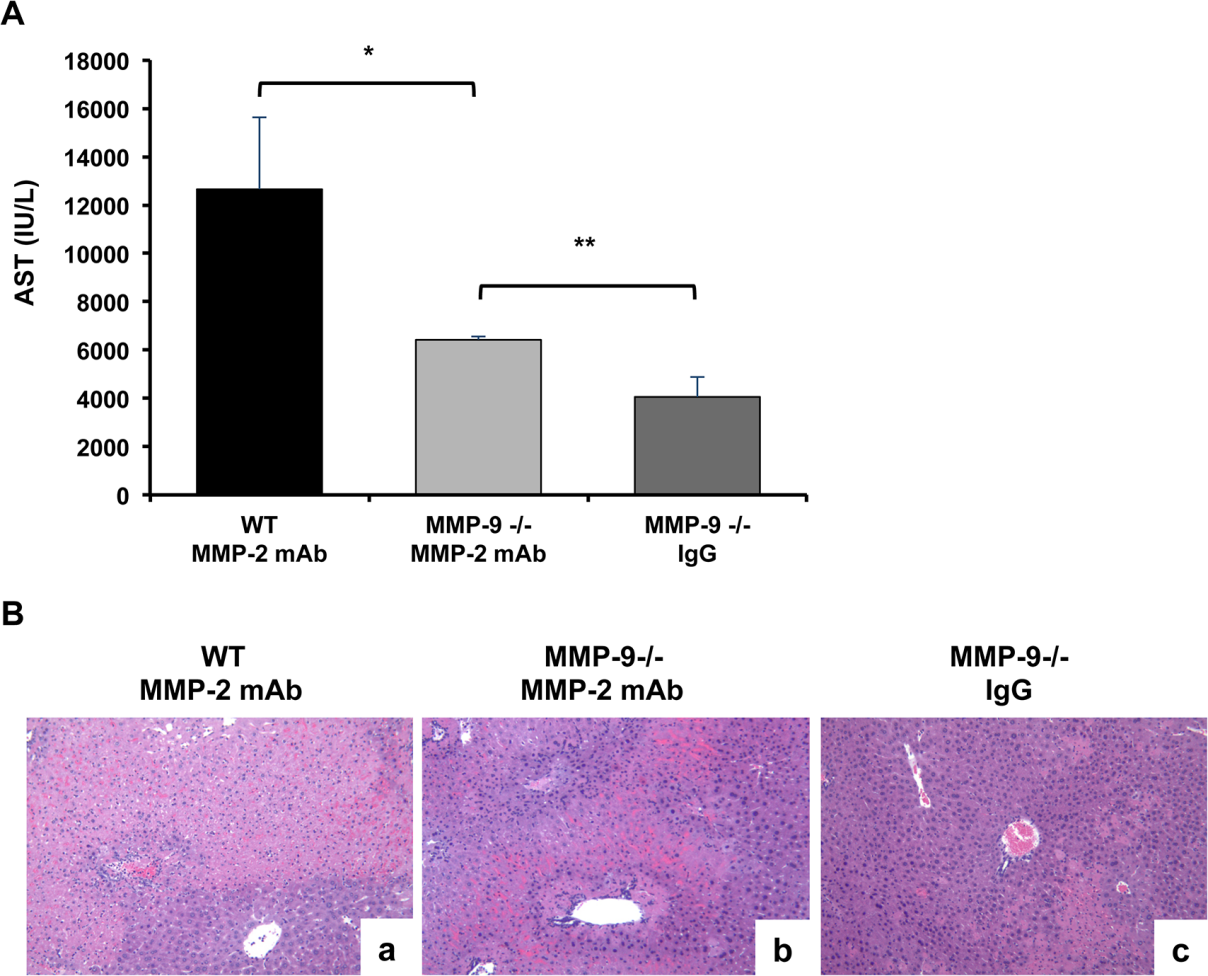
Anti-MMP-2 antibody therapy in MMP-9 null mice. The serum AST levels (panel A) in MMP-9^-/-^ mice treated with anti-MMP-2 antibodies at 6h of liver IRI were significantly reduced when compared to wild-type mice treated with anti-MMP-2 antibodies and increased when compared to MMP-9^-/-^ mice treated with IgG. Histological preservation (panel B) was markedly improved in MMP-9 null livers treated with MMP-2 mAb at 6h after IRI (b), when compared to the extensively damaged anti-MMP-2 mAb treated WT livers (a), but inferior when compared to MMP-9^-/-^ livers treated with control IgG (c) (n = 4/group; *p<0.05 relative to WT+MMP-2mAb; **p<0.05 relative to MMP-9^-/-^+IgG).

In an additional series of experiments, MMP-9 null mice were treated with neutralizing anti-MMP-2 antibodies to assess whether MMP-9 deficiency would confer protection against MMP-2 mAb-mediated liver damage. Indeed, MMP-2 mAb-induced liver damage was markedly reduced in MMP-9 null mice post-liver IRI; MMP-9^-/-^ mice treated with anti-MMP-2 antibodies were characterized by significantly lower AST levels and improved liver histological preservation after reperfusion when compared to wild-type littermates treated with anti-MMP-2 antibodies (Fig 9). However, MMP-9 null mice treated with anti-MMP-2 antibodies showed more liver damage than MMP-9 null mice treated with IgG, (Fig 9). All together, these results support an important, but not exclusive, mechanism in which MMP-2 specific inhibition amplifies MMP-9 activity and worsens hepatic IRI.

## Discussion

The expression of gelatinases (MMP-2 and MMP-9) has been linked to a wide range of pathological conditions, including hepatic IRI [3]. Indeed, given the significant role that gelatinases may have in disease progression, these MMPs have long been drug targets [22]. However, MMPs can also act on physiological substrates and their inhibition can potentially be detrimental [6]. Moreover, the same MMP can have opposing functions dependent on the context of disease progression [17]. It is therefore important to understand the individual roles of MMPs during hepatic IRI in order to assist in the design of novel therapies that target pro-inflammatory MMPs while sparing MMPs with potential protective roles [31].

In this study, we used MMP-2-deficient mice and their respective wild-type littermates to examine the direct function of MMP-2 expression in the development of hepatic IRI. MMP-2 expression was detected along the sinusoids of MMP-2^+/+^ livers prior and after IRI. Additionally, a relatively small population of infiltrating leukocytes, predominantly neutrophils, was also positive for MMP-2 in wild-type livers post-reperfusion. When compared to the already significantly injured MMP-2^+/+^ control livers, MMP-2^-/-^ deficient mice had exacerbated liver damage after hepatic IRI. The more severe liver lesions caused by MMP-2 gene deletion likely contributed to the critically compromised liver function and animal loss, which occurred after the second day post-reperfusion. This is particularly relevant in that the model of partial liver IRI is nonlethal [32]. MMP-2^-/-^ livers after IRI were characterized by massive accumulation of infiltrating leukocytes and increased levels of pro-inflammatory cytokines, such as IFN-γ, TNF-α, and IL-6. Our observations that the lack of MMP-2 worsened liver damage are also supported by our own studies with an anti-MMP-2 antibody, which aggravated mouse liver IRI. Therefore, in contrast to MMP-9 deletion, which exerts protection against the liver IR-insult [11,33], complete loss of MMP-2 activity promoted inflammatory responses in hepatic IRI. Paradoxically, MMP-2 ablation can have both beneficial and detrimental roles, depending on the etiology of the tissue damage [34]. In line with our observations, a protective role for MMP-2 expression has been reported in experimental models of antibody-induced arthritis, autoimmune encephalomyelitis, and myocarditis [14,15,20,35,36]. Moreover, MMP-2 increased activity has also been associated to the beneficial effects of preconditioning in reduced-size orthotopic liver transplantation [37]. Here, for the first time, we show the role of complete and specific MMP-2 activity suppression in hepatic IRI. Current pharmacological MMP inhibitors suitable for *in vivo* use differ in their inhibitory potencies towards MMPs, but none of these drugs is selective for a given MMP [22]. Additionally, the applicability of commercially available MMP inhibitors to *in vivo* studies has potential limitations; there is still significant lack of information on the *in vivo* efficacy and specificity of these drugs, which are frequently unstable in solution and require the use of DMSO or ethanol as solvents. These solvents are apparently innocuous at concentrations required to dissolve small amounts of inhibitor typically used *in vitro*, but they could potentially mask the effect of the inhibitor *in vivo* as they have dose-dependent adverse outcomes [38,39]. Taken together, gene manipulation, rather than drug-mediated MMP inhibition, is presently the most effective way to evaluate the contribution of a specific MMP and to obtain direct evidence of its role [40].

Leukocyte migration across vascular and extracellular matrix (ECM) barriers is essential for assembling a successful inflammatory response [3]. We and others have reported a central role for leukocyte-expressed MMP-9 on cellular transmigration and activation leading to liver injury [3]. In this study, MMP-2 depletion resulted in increased MMP-9 activity, spontaneous leukocyte infiltration in naïve livers, and massive numbers of MMP-9+ leukocytes in damaged livers post-reperfusion. Neutrophils are particularly detrimental in hepatic IRI [41]. Mechanistically, using selective MMP inhibitors suitable for *in vitro* testing, our studies show that MMP-9 activity was upregulated in both MMP-2^+/+^ neutrophils treated with a MMP-2 inhibitor and MMP-2^-/—^null neutrophils, which correlated with the increased transmigration capabilities of these cells. We have previously shown that MMP-9 facilitates neutrophil migration and that MMP-9^-/—^null neutrophils have reduced transmigration activity [11]. Indeed, inhibition of MMP-9 activity significantly depressed the transmigration of MMP-2^-/—^null neutrophils. Moreover, administration of anti-MMP-2 neutralizing antibodies to MMP-9 null mice resulted in significantly reduced liver damage post IRI when compared to wild-type mice treated with anti-MMP-2 antibodies, confirming an important mechanism of action for MMP-2 via regulation of MMP-9 activity. An increase of MMP-9 expression in the absence of MMP-2 activity was previously observed in experimental autoimmune encephalomyelitis [36]. Therefore, similarly to what was observed in experimental autoimmune encephalomyelitis, our data suggest that loss of MMP-2 activity in hepatic IRI exacerbated disease, in part, by augmenting MMP-9 secretion. The regulation of MMP activity is very complex; it occurs at various levels and can be mediated by several proteinases, including active MMPs [5]. Further studies are needed to determine the mechanisms underlying induction of MMP-9 expression by loss of MMP-2 activity during hepatic IRI.

MMPs can act on different substrates and have multifactorial roles in a particular tissue [6]. It has been demonstrated that active MMP-2, which is constitutively present in the nuclei of endothelial cells [42], degrades PARP-1 *in vitro* [26]. PARP-1 is a key mediator of liver inflammation [43]. Loss of MMP-2 activity resulted in a profound reduction of the cleaved PARP/full-length PARP ratios after hepatic IRI, suggesting that PARP-1 is also cleaved by MMP-2 in our *in vivo* settings. Moreover, the ratios of cleaved PARP/PARP were markedly decreased in lysates of MMP-2^-/—^null liver sinusoidal endothelial cells and in lysates of MMP-2^+/+^ liver sinusoidal endothelial cells treated with a specific MMP-2 inhibitor, supporting our *in vivo* observations. The reduction in PARP-1 proteolytic degradation observed in MMP-2 null liver sinusoidal endothelial cells and in MMP-2 inhibitor-treated MMP-2^+/+^ liver sinusoidal endothelial cells correlated with the increased cytotoxicity of these cells, which was reversed by the addition of a PARP-1 selective inhibitor. Indeed, it has been demonstrated that failure to degrade excessive PARP-1 causes cell death after IRI [44]. Furthermore, the exogenous administration of the PARP-1 selective inhibitor in MMP-2 null mice resulted in lower MMP-9 activity levels and in a fairly modest but significant protection again hepatic IRI. Thus, these results confirm previous findings of PARP-1 being a substrate of MMP-2 and suggest a protective PARP-1 degradation-dependent role of MMP-2 activity in hepatic IRI.

Our study provides direct evidence of a protective role for MMP-2 expression in liver and offers new insights into MMP modulation of inflammatory/immune responses in hepatic IRI. Complete loss of MMP-2 activity led to exacerbated tissue damage, massive leukocyte infiltration, extensive necrosis and animal death after liver IRI. MMP-2 gene deletion resulted in enhanced MMP-9 activity, spontaneous leukocyte infiltration in naïve liver, and amplified MMP-9+ leukocyte recruitment and activation post-liver reperfusion. Additionally, MMP-2 inhibition impaired the degradation of PARP-1 in isolated sinusoidal endothelial cells and in liver IRI. All together, these results support the view that the future design of improved pharmacological MMP-targeted agents to treat hepatic IRI should spare the activity of MMP-2 in the liver. Moreover, they stress the need of innovative chemistries to generate specific inhibitors of individual MMPs that spare MMPs with beneficial actions [45].
